# Normative Data and Minimally Detectable Change for Inner Retinal Layer Thicknesses Using a Semi-automated OCT Image Segmentation Pipeline

**DOI:** 10.3389/fneur.2019.01117

**Published:** 2019-11-25

**Authors:** Seyedamirhosein Motamedi, Kay Gawlik, Noah Ayadi, Hanna G. Zimmermann, Susanna Asseyer, Charlotte Bereuter, Janine Mikolajczak, Friedemann Paul, Ella Maria Kadas, Alexander Ulrich Brandt

**Affiliations:** ^1^NeuroCure Clinical Research Center, Charité-Universitätsmedizin Berlin, Corporate Member of Freie Universität Berlin, Humboldt-Universität zu Berlin, and Berlin Institute of Health, Berlin, Germany; ^2^Experimental and Clinical Research Center, Max Delbrück Center for Molecular Medicine and Charité-Universitätsmedizin Berlin, Corporate Member of Freie Universität Berlin, Humboldt-Universität zu Berlin, and Berlin Institute of Health, Berlin, Germany; ^3^Department of Neurology, Charité-Universitätsmedizin Berlin, Corporate Member of Freie Universität Berlin, Humboldt-Universität zu Berlin, and Berlin Institute of Health, Berlin, Germany; ^4^Department of Neurology, University of California, Irvine, Irvine, CA, United States

**Keywords:** optical coherence tomography (OCT), retina, normative data, inner retinal layer, segmentation, macula, healthy population, minimally detectable change

## Abstract

Neurodegenerative and neuroinflammatory diseases regularly cause optic nerve and retinal damage. Evaluating retinal changes using optical coherence tomography (OCT) in diseases like multiple sclerosis has thus become increasingly relevant. However, intraretinal segmentation, a necessary step for interpreting retinal changes in the context of these diseases, is not standardized and often requires manual correction. Here we present a semi-automatic intraretinal layer segmentation pipeline and establish normative values for retinal layer thicknesses at the macula, including dependencies on age, sex, and refractive error. Spectral domain OCT macular 3D volume scans were obtained from healthy participants using a Heidelberg Engineering Spectralis OCT. A semi-automated segmentation tool (SAMIRIX) based on an interchangeable third-party segmentation algorithm was developed and employed for segmentation, correction, and thickness computation of intraretinal layers. Normative data is reported from a 6 mm Early Treatment Diabetic Retinopathy Study (ETDRS) circle around the fovea. An interactive toolbox for the normative database allows surveying for additional normative data. We cross-sectionally evaluated data from 218 healthy volunteers (144 females/74 males, age 36.5 ± 12.3 years, range 18–69 years). Average macular thickness (MT) was 313.70 ± 12.02 μm, macular retinal nerve fiber layer thickness (mRNFL) 39.53 ± 3.57 μm, ganglion cell and inner plexiform layer thickness (GCIPL) 70.81 ± 4.87 μm, and inner nuclear layer thickness (INL) 35.93 ± 2.34 μm. All retinal layer thicknesses decreased with age. MT and GCIPL were associated with sex, with males showing higher thicknesses. Layer thicknesses were also positively associated with each other. Repeated-measurement reliability for the manual correction of automatic intraretinal segmentation results was excellent, with an intra-class correlation coefficient >0.99 for all layers. The SAMIRIX toolbox can simplify intraretinal segmentation in research applications, and the normative data application may serve as an expandable reference for studies, in which normative data cannot be otherwise obtained.

## 1. Introduction

Optical coherence tomography (OCT) allows non-invasive high-resolution *in vivo* imaging of the retina (1). Spectral domain OCT (SD-OCT) provides 3D volume scans of the retina, and intraretinal segmentation of macular volume scans enables quantitative OCT applications in neurodegenerative and autoimmune neuroinflammatory disorders (2, 3). The inner retinal layers, in particular, are currently of pivotal interest for several neurologic disorders. For example, the combined macular ganglion cell and inner plexiform layer (GCIPL) thickness reflects disease severity and activity in patients with multiple sclerosis (MS) (4) and is suggested for monitoring disease activity in MS (5). GCIPL might further serve to identify neurodegeneration already very early on in the disease (6), and could thus be used as a marker for assessing the individual risk of a patient at onset for an active disease course (7). GCIPL is also suggested as a sensitive marker for attack severity in acute optic neuritis (8, 9). The inner nuclear layer (INL), on the other hand, is a marker for inflammatory disease activity in MS and might be utilized to monitor treatment response (10–12). In neuromyelitis optica spectrum disorders (NMOSD), the INL might be affected as part of an autoimmune reaction against Müller cells (13), which could lead in turn to progressive GCIPL loss (14).

Intraretinal layer segmentation is a crucial step in measuring GCIPL or INL changes. In recent years, many algorithms for intraretinal layer segmentation have been developed, and are now routinely implemented in clinical OCT devices or are available as external tools for research (15). While reliability in healthy eyes is usually good (16), many scans in diseases with macroscopic retinal changes or signal quality issues caused by more difficult OCT measurement in vision-impaired individuals require quality control and manual correction (17). Proper user interfaces for manual correction of automatic segmentation results are not always available, having led to many studies with questionable OCT data based on very small regions of interest (6) or inappropriate quality control (17).

Many studies have investigated intraretinal layer thicknesses in healthy eyes to establish normative reference values, recently e.g., Invernizzi et al. (18). Clinical features like age, sex, and axial length have been reported to physiologically affect intraretinal layer thicknesses (18, 19). But normative data studies are often only applicable in a narrow context depending on the selected samples and the methodology used, and data from studies from Asia, or as a control for different diseases, are not necessarily applicable in the context of neuroinflammatory diseases in European or North American populations.

In this study we aimed (a) to establish normative values for inner intraretinal layer thicknesses in a healthy Caucasian population and age/sex distribution suitable for typical autoimmune neuroinflammatory disorders, and (b) to evaluate layer thicknesses in association with age and sex. For this task we developed an easily usable and adaptable intraretinal segmentation pipeline based on an interchangeable third-party segmentation algorithm (20) as well as a survey tool for additional normative data, which together allow data surveys also beyond the scope of this study. Both are made available as an open source application along with this publication.

## 2. Materials and Methods

### 2.1. Study Population

We queried our institute's research database to create a normative OCT database. The database contained healthy control data from two multimodal register studies aiming to evaluate quantitative measurements of neuro-axonal damage in MS and other neuroinflammatory disorders who were recruited from July 2010 to March 2018 at the NeuroCure Clinical Research Center at the Charité-Universitätsmedizin Berlin. Each participant underwent an examination of both eyes with Spectralis SD-OCT. Retrospective inclusion criteria for the present study were participants in a healthy condition aged between 18 and 70 years, Caucasian ethnicity, and high-quality macular OCT scans (signal strength more than 15 dB). Exclusion criteria were any neurological condition, any other disorder known to affect the retina (i.e., diabetes), any eye disease affecting the retina (i.e., glaucoma), any relevant pathological finding in the neurovisual examination performed by experienced optometrists, and a refractive error above ±6 diopters. Twenty high quality macular OCT scans (signal strength more than 15 dB) of NMOSD patients all with the history of optic neuritis (ON) were randomly selected from our database to test the performance of the segmentation pipeline presented in this study.

The study was approved by the ethics committee of Charité–Universitätsmedizin Berlin and conducted according to the Declaration of Helsinki in its currently applicable version. All participants gave written informed consent.

### 2.2. Optical Coherence Tomography

All OCT measurements were carried out with a Spectralis SD-OCT and Heidelberg Eye Explorer (HEYEX) version 5.7.5.0 (Heidelberg Engineering, Heidelberg, Germany), by eight individual operators, with automatic real-time (ART) function for image averaging and an activated eye tracker in a dimly lit room. Macular 3D volumes were assessed by a custom scan comprising 61 vertical B-scans (each with 768 A-Scans, with ART of 13 frames) with a scanning angle of 30 × 25° focusing on the fovea. All scans were quality controlled according to the OSCAR-IB criteria (21) and reporting adheres to APOSTEL recommendations (22). Scans not passing the quality control were excluded from analysis.

The macular scans were exported from the device and stored in HEYEX Vol file format (*.vol files), and then intraretinal segmentation was performed using the segmentation pipeline as described below. All segmentation results were quality controlled and manually corrected in case of errors by an experienced grader. In the end, the thickness data was calculated and stored in a CSV file format (.csv) for further analysis. The Early Treatment Diabetic Retinopathy Study (ETDRS) macular map, as described by the ETDRS research group (23), were used for this study. We report average macular thickness (MT), macular retinal nerve fiber layer thickness (mRNFL), combined ganglion cell and inner plexiform layer thickness (GCIPL), and inner nuclear layer thickness (INL) in the entire ETDRS macular map (the 6 mm diameter circular area around the fovea). Other layer thicknesses (e.g., outer retinal layers) and the thicknesses in different sectors of the ETDRS macular map can be studied using the provided *shiny* application and source data (Supplementary Material).

### 2.3. Intraretinal Segmentation

**Figure 1:**
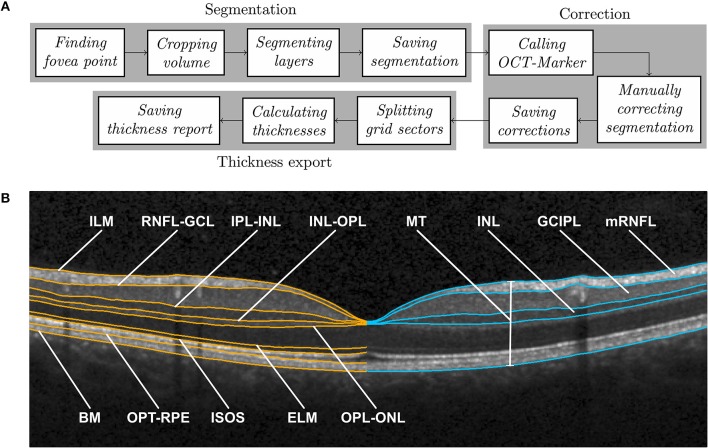
**(A)** SAMIRIX pipeline, and **(B)** Boundaries delineated by layer segmentation (in orange) on the left and derived layers with manually corrected boundaries (in blue) on the right, shown on a central B-scan crossing the fovea. ILM, inner limiting membrane; RNFL, retinal nerve fiber layer; GCL, ganglion cell layer; IPL, inner plexiform layer; INL, inner nuclear layer; OPL, outer plexiform layer; ONL, outer nuclear layer; ELM, external limiting membrane; ISOS, inner and outer segments; OPT, outer photoreceptor tips; RPE, retinal pigment epithelium; BM, Bruch membrane; MT, macular thickness; mRNFL, macular retinal nerve fiber layer; GCIPL, combined ganglion cell and plexiform layer; INL, inner nuclear layer.

Intraretinal segmentation, manual correction, and thickness data export of all macular scans were done using a custom-developed intraretinal segmentation pipeline (SAMIRIX). SAMIRIX modularly includes import filters for OCT data, a third-party segmentation algorithm, a user interface for controlling and correcting segmentation results, and batch-operations for processing multiple OCT images (Figure 1A).

SAMIRIX was developed in MATLAB (R2017a, MathWorks, Natick, MA, USA) and the user interface OCT-Marker was written in C++11, by using Qt5, Boost, and OpenCV libraries.

#### 2.3.1. Segmentation Algorithm

As a segmentation algorithm we used OCTLayerSegmentation (20), which has been released as a package of AURA Tools on NITRC (https://www.nitrc.org/projects/aura_tools/). We chose OCTLayerSegmentation, because it showed good performance and accuracy with an overall absolute error of 3.5 μm by combining a machine learning approach for boundary classification (random forest classification) and a robust state-of-the-art graph-cut algorithm boundary refinement (optimal graph search) in a previous study (24). OCTLayerSegmentation delineates the inner limiting membrane (ILM), external limiting membrane (ELM), Bruch membrane (BM), and the boundaries between the retinal nerve fiber layer and ganglion cell layer (RNFL-GCL), inner plexiform layer and inner nuclear layer (IPL-INL), inner nuclear layer and outer plexiform layer (INL-OPL), outer plexiform layer and outer nuclear layer (OPL-ONL), inner and outer segments (ISOS), and outer photoreceptor tips and retinal pigment epithelium (OPT-RPE) (Figure 1B). These boundaries then serve to calculate intraretinal layer areas with nomenclature as suggested by the APOSTEL criteria (22).

For segmentation, the first step is to automatically find the central fovea point of the macular volume scan to be segmented. Based on the segmentation of the ILM and BM by the Heidelberg Engineering Eye Explorer (HEYEX) software, the height difference between the two layers is computed. In order to detect the lowest point of the foveal surface, we look at the minimum of this difference within the 1 mm circular area around the center automatically defined by HEYEX. If several minima are detected, then the median point of them is taken as the center of the foveal pit. The next step is to crop the volume to 6–6 mm square around the fovea, aligned with the main direction of the scan. This was done because many segmentation approaches work with a priori assumption regarding the expected image. The algorithm by Lang et al. used in this version of SAMIRIX works well with this volume, which was also used by the original developers of the algorithm (20). After being cropped, the volume is segmented by the integrated 3rd-party segmentation algorithm (20). The segmentation results are then read by SAMIRIX and saved alongside the volume in a single file.

#### 2.3.2. User Interface for Manual Correction

For quality control and manual correction, we developed a graphical user interface (OCT-Marker). In the first step, the scan to be checked and corrected if necessary, is opened in OCT-Marker. A Piecewise Cubic Hermite Interpolating Polynomial (PCHIP) based correction method with defined control points is provided to the user to ease the correction process. This enables modifications on the segmentation results while going through the volume scan, B-scan by B-scan. When the correction is done, the modified segmentation is written and saved over the previous one in the data file.

#### 2.3.3. Data Export and Batch Processing

For thickness data export, the user selects the upper and lower boundaries of the layer, and also the grid in which the thickness is going to be calculated (e.g., ETDRS 6 mm grid). Then, each volume is split into these sectors, and the average thickness of each is computed. At the end, the calculated values are written and saved in a comma separated values (csv) file.

SAMIRIX also offers the possibility of performing batch segmentation. For this purpose, the selected volumes are taken through the steps in the segmentation module, one by one. Also, in the thickness export module, the first two steps are repeated for each volume, and then the end result consists in a single thickness report saved in a single table. SAMIRIX only works with Spectralis OCT scans in HEYEX Vol file format (*.vol files). Screenshots from SAMIRIX and OCT-Marker are provided in Supplementary Material.

### 2.4. Statistical Analysis

Statistical analysis was done in R [Version 3.4.4 (25)]. Exploratory data analysis and data visualization were performed using the ggplot2 package (26). For assessment of consistency, the intra-class correlation coefficient (ICC) and 95% confidence intervals were estimated using the ICC package (27), based on the variance components from a one-way ANOVA. The coefficient of variation (CV), standard error of measurement (SEM), and minimum detectable change (MDC) for inter-rater and intra-rater consistency analysis were calculated based on the formulas described by Beckerman et al. (28). SEM and MDC, the latter sometimes also called smallest real difference (SRD), are statistical approaches to estimate the minimally needed difference between two measurements that a method is able to detect (28), and is used in this study as a measure to quantify the amount of noise. In this study, an ICC >0.9 was considered as high, between 0.8 and 0.9 as moderate, and <0.8 as insufficient, as suggested by Vaz et al. (29).

Analysis of OCT values against age, sex, and refractive error was performed by linear mixed effect models (LMM), including inter-eye within-patient correlations as a random effect [lme4 package (30), and lmerTest package (31)]. The conditional and marginal coefficients of determination were calculated with pseudo R-squared [MuMIn package (32)]. The correlation of OCT values was assessed using Pearson's product-moment correlation [stats package (25)] and regression analysis was carried out using LMM with the inclusion of inter-eye within-patient correlations as a random effect. For this study, *p*-values below 0.05 were considered significant.

All statistical and exploratory results of this study were established in an interactive HTML document using R Markdown (33) and Shiny (34) packages. R Markdown is a framework to run codes written in R and generates reports based on the output of the codes. By using Shiny R package, the reports can be turned into interactive web applications. The documents based on R Markdown and Shiny packages can be deployed on web servers and are therefore accessible, like web pages. A screenshot of the interactive HTML document is provided in the Supplementary Material.

## 3. Results

Initially, macula scans of 438 eyes of 219 subjects were collected from our database according to the inclusion and exclusion criteria, from which the scans from 15 eyes of 14 subjects were excluded due to insufficient scan quality. Therefore, in this study, macula scans of 423 eyes of 218 subjects of Caucasian descent were included, from which 144 (66 %) subjects were females and 74 (34 %) were males. Age ranged between 18 and 69 years, with an average [±standard deviation (SD)] of 36.5 ± 12.27 years. Refractive error was available from a subset of 70 eyes (35 subjects), from which the average was −0.55 ± 1.38 SD diopter with a range between −4.75 and +1.75 diopter.

**Figure 2:**
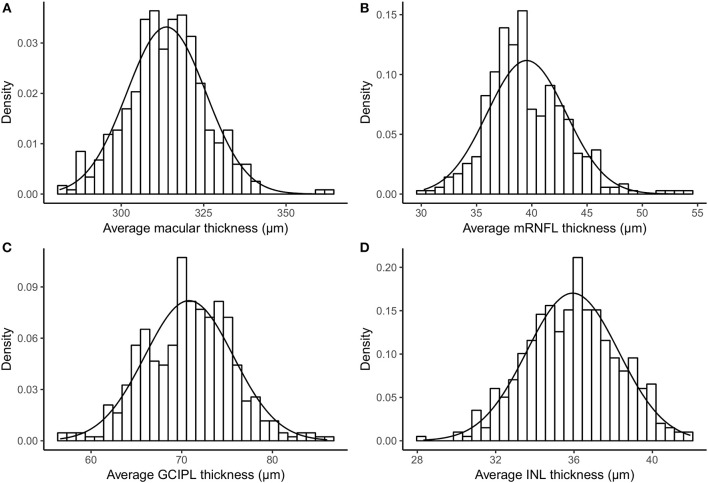
Histogram and fitted normal distribution curve of average thickness of **(A)** macula, **(B)** mRNFL, **(C)** GCIPL, and **(D)** INL. mRNFL, macular retinal nerve fiber layer; GCIPL, combined ganglion cell and plexiform layer; INL, inner nuclear layer.

Table 1 provides descriptive statistics of average MT, mRNFL, GCIPL, and INL thicknesses, including the mean, SD, coefficient of variation, range, first percentile, fifth percentile, ninety-fifth percentile, and ninety-ninth percentile. Figure 2 shows the distribution of the average layer thicknesses together with an overlaid curve representing normal distribution fitted to each graph.

**Table 1:** Descriptive statistics of average thicknesses in the entire ETDRS macular map.

**Average thickness (μm)**	**Mean ±SD**	**CV (%)**	**Min–Max**	**1st–99th percentile**	**5th–95th percentile**
MT	313.70 ± 12.02	3.83	281.29–362.29	286.53–339.59	294.20–333.25
mRNFL	39.53 ± 3.57	9.03	30.22–54.38	32.41–49.18	34.35–45.68
GCIPL	70.81 ± 4.87	6.87	56.60–86.03	59.00–83.56	63.16–77.95
INL	35.93 ± 2.34	6.52	28.31–41.94	31.00–40.97	32.08–39.87

*MT, macular thickness; mRNFL, macular retinal nerve fiber layer; GCIPL, combined ganglion cell and plexiform layer; INL, inner nuclear layer; CV, coefficient of variation; Min, minimum; Max, maximum*.

**Figure 3:**
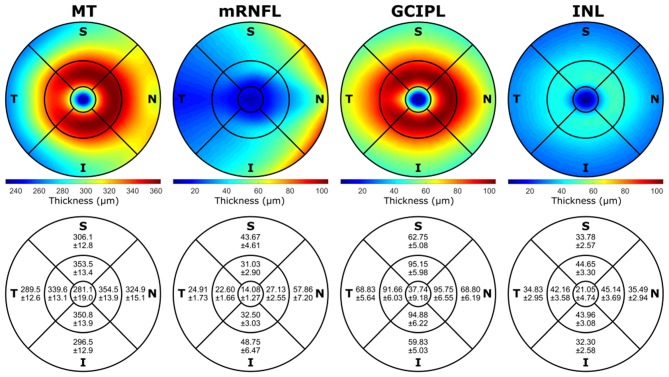
The mean of the layer thicknesses of all the volumes included in this study in the ETDRS macular map area shown as heat maps, together with the mean and standard deviation (Mean±SD) of the average layer thicknesses in different sectors of the ETDRS macular map. The mRNFL, GCIPL, and INL heat maps have the same scale with the minimum and maximum values of all the three layers (min: 6.28 μm, max: 104.02 μm) in the interest of comparison. The left eyes were mirrored along the vertical axis, and all the images were rotated to be aligned with the fovea and center of the optic nerve head axis. All the numbers are in micrometers. MT, macular thickness; mRNFL, macular retinal nerve fiber layer; GCIPL, combined ganglion cell and plexiform layer; INL, inner nuclear layer; T, temporal; N, nasal; S, superior; I, inferior; ETDRS, Early Treatment Diabetic Retinopathy Study; Min, minimum; Max, maximum.

Additionally, the normative (mean) thickness of the MT, mRNFL, GCIPL, and INL layers in the ETDRS macular map is shown as heat maps in Figure 3, alongside the normative values of the average layer thicknesses of the eyes included in this study in the ETDRS macular map sectors. Descriptive statistics of the layer thicknesses in the ETDRS macular map sectors is provided in Supplementary Material.

To test inter-rater reliability, the automatic segmentation results of 44 eyes of 24 subjects from this study were manually corrected by two different experienced graders, who were masked. We then calculated the intra-class correlation coefficient (ICC) and minimum detectable change (MDC) for MT, mRNFL, GCIPL, and INL, which is detailed in Table 2.

**Table 2:** Inter-rater reliability measurements of segmentation corrections.

**Average thickness (μm)**	**ICC**	**Upper CI**	**Lower CI**	**CV**	**SEM**	**MDC**
MT	0.99984	0.99971	0.99991	0.04464	0.13728	0.38052
mRNFL	0.99350	0.98817	0.99644	0.57594	0.23771	0.65889
GCIPL	0.99794	0.99625	0.99887	0.21960	0.16519	0.45788
INL	0.99734	0.99515	0.99854	0.27271	0.10564	0.29281

*MT, macular thickness; mRNFL, macular retinal nerve fiber layer; GCIPL, combined ganglion cell and plexiform layer; INL, inner nuclear layer; ICC, intra-class correlation coefficient; CI, confidence interval; CV, coefficient of variation; SEM, standard error of measurement; MDC, minimum detectable change*.

Intra-rater reliability of the manual correction was tested by manually correcting the segmentation results of the same set of OCT scans from the previous reliability test (44 eyes of 24 subjects) twice by an experienced grader. The MDC (and ICC) was 0.24 (0.99994), 0.31 (0.99861), 0.23 (0.99947), and 0.19 micrometers (0.99890) for MT, mRNFL, GCIPL, and INL, respectively.

Regression analysis of layer thicknesses against age showed significant changes. In particular, MT showed an average decrease of 0.215 μm per year (*p*-value = 0.001). Likewise, GCIPL thickness decreased by on average 0.088 μm per year (*p*-value = 0.001). Significant changes of average thickness of mRNFL and INL by aging are also reported; Table 3 provides detailed results.

**Table 3:** Regression analysis of average thicknesses against age, sex, and refractive error.

**Average thickness (μm)**	**Against**	**Mean (SD)**	**B**	**SE**	***P***	**${\text{R}}_{\text{M}arg.}^{2}$**	**${\text{R}}_{\text{C}ond.}^{2}$**
MT	Age (years)		−0.2148	0.0648	**0.0010**	0.0478	0.9679
	Sex: F vs. M	312.32 (12.11) 316.38 (11.41)	4.1771	1.6941	**0.0145**	0.0268	0.9679
	RE (diopter)		−0.3926	0.7013	0.5777	0.0030	0.9644
mRNFL	Age (years)		−0.0523	0.0192	**0.0072**	0.0312	0.8821
	Sex: F vs. M	39.46 (3.69) 39.67 (3.33)	0.3152	0.5056	0.5337	0.0017	0.8820
	RE (diopter)		−0.6067	0.2936	**0.0430**	0.0729	0.8753
GCIPL	Age (years)		−0.0874	0.0263	**0.0010**	0.0480	0.9652
	Sex: F vs. M	70.28 (4.98) 71.82 (4.48)	1.5150	0.6896	**0.0291**	0.0214	0.9652
	RE (diopter)		0.0049	0.2950	0.9869	0	0.9730
INL	Age (years)		−0.0453	0.0125	**0.0004**	0.0558	0.9331
	Sex: F vs. M	35.82 (2.40) 36.15 (2.22)	0.3526	0.3312	0.2883	0.0050	0.9331
	RE (diopter)		0.2394	0.1783	0.1841	0.0209	0.9512

*MT, macular thickness; mRNFL, macular retinal nerve fiber layer; GCIPL, combined ganglion cell and plexiform layer; INL, inner nuclear layer; F, female; M, male; vs., versus; RE, refractive error; SD, standard deviation; B, slope; SE, standard error of B; P, p-value; ${\text{R}}_{Marg.}^{2}$, Marginal R-squared; ${\text{R}}_{Cond.}^{2}$, Conditional R-Squared. Significant p-values marked in bold*.

Analysis of average layer thicknesses vs. sex revealed significant differences in MT and GCIPL between males and females. Males showed on average 4.18 μm higher MT than females (*p*-value = 0.015). Further, males had a 1.52 μm thicker GCIPL in comparison to females (*p*-value = 0.029). As reported in Table 3, neither mRNFL nor INL thickness showed significant sex differences.

**Figure 4:**
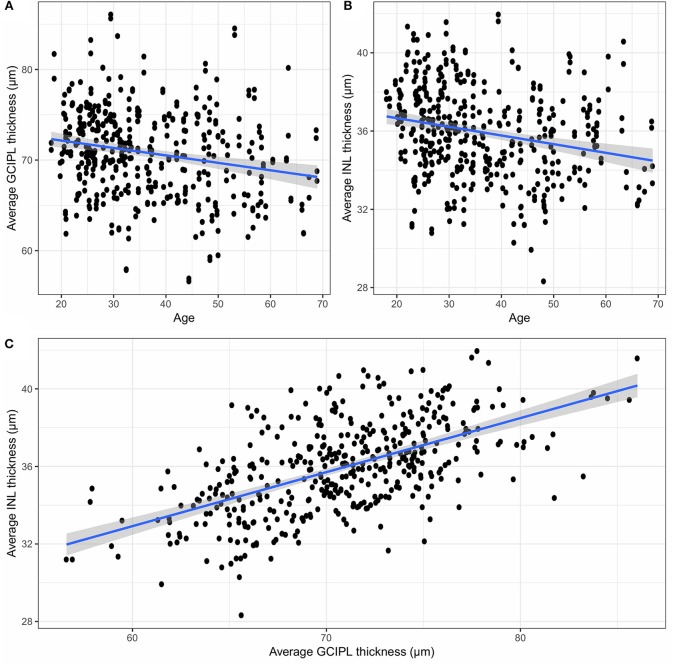
**(A)** The average GCIPL thickness against age, **(B)** the average INL thickness against age, and **(C)** the average INL thickness against the average GCIPL thickness. GCIPL, combined ganglion cell and plexiform layer; INL, inner nuclear layer.

Since GCIPL and INL are of particular interest, Figure 4 shows the average GCIPL and INL thicknesses against age. The INL thickness was also plotted against the GCIPL thickness in Figure 4. The correlation coefficient between the INL and GCIPL thicknesses was 0.579 (*p*-value < 2 × 10^−16^) and the slope (B) of the linear regression was 0.277 [standard error (SE) = 0.022, *p*-value < 2 × 10^−16^].

To test the performance of SAMIRIX and to compare it with the performance of the HEYEX software, 20 OCT scans from NMOSD patients, all with a history of optic neuritis (ON) were segmented, and the segmentation results were manually corrected by a grader experienced in both SAMIRIX and HEYEX. The median correction time for SAMIRIX was 7:59 min (minimum: 5:07 min, maximum: 27:22 min), while the median correction time for HEYEX was 10:30 min (minimum: 8:01 min, maximum: 22:01 min). The mean absolute correction in the 6 mm ETDRS circle (the amount of correction for all the five corrected boundaries ILM, RNFL-GCL, IPL-INL, INL-OPL, and BM divided by the number of A-Scans in the 6 mm ETDRS circle) was also calculated. For the mean absolute correction in SAMIRIX, the median was 0.16 μm (minimum: 0 μm, maximum: 22.45 μm), and in HEYEX, the median was 0.79 μm (minimum: 0.06 μm, maximum: 2.02 μm).

## 4. Discussion

In this study we present normative data for inner intraretinal layer thicknesses of a large cohort of 218 healthy subjects (423 eyes) of Caucasian ethnicity aged between 18 and 69 years, using Spectralis SD-OCT 3D macular scans.

In our study the average thickness of all investigated layers was associated with age, which is consistent with other studies (35–40). Recently, Invernizzi et al. (18) investigated the association of different intraretinal layer thicknesses in the outer and middle rings and the center of the ETDRS thickness map, with age, and showed no significant association in any regions except the center of macular thickness, which is consistent with some other studies (41, 42). von Hanno et al. (43) suggested a positive association between macular thickness and age up to around 60 years and a negative association afterwards, by studying retinal OCTs of 4,508 eyes. Previous studies investigating retinal thicknesses in relation to sex in a healthy population showed that women had thinner retinal thickness measures than men (36, 40, 41, 43, 44). Our results are in accordance with this for MT and GCIPL, but not for mRNFL and INL, which were both not sex-dependent in our cohort. The analysis using OCT data from the UK Biobank study (67,321 adults) from (45) reported associations among older age, ethnicity, BMI, smoking, and macular thickness.

Inter-rater reliability of manually corrected segmentation results was excellent with ICC values above 0.99 for all layers. MDC, from the inter-rater reliability test, was 0.46 μm for GCIPL, which is higher than the projected annual loss in healthy subjects in this study (0.09 μm per year) and similar to the average annual GCIPL loss reported in patients with MS (−1.1 μm over 2 years) (46). This means that current intraretinal segmentation is not able to reliably detect annual GCIPL loss in an individual MS patient, and further technological improvements in acquisition and image analysis are required to allow this, e.g., for clinical monitoring applications. Intraretinal segmentation of the GCIPL is, however, suited to track optic neuritis associated damage, which is often magnitudes higher than the observed MDC in this study (8, 9). For INL, the inter-rater MDC in our study was 0.29 μm, which is similar to group-wise changes reported with disease activity related effects in the range of 0.35 to 0.71 μm (12). Again, this suggests that current OCT intraretinal segmentation is not able to reliably detect meaningful INL change for this application. A previous multicenter study using the device's own semi-automatic segmentation approach with manual correction produced even higher MDC (17). While previous studies, and our current study only investigated segmentation-based reliability on a single scan, the additional acquisition noise from two different scans is likely to result in even higher MDC in a real world scenario of follow-up measurements. The reported MDC is below the resolution of the used SD-OCT technology, which suggests that imaging rather than segmentation is the limiting issue in detecting change.

To further support the opposing roles of GCIPL and INL measurements in neuroinflammatory disorders, we investigated their association in healthy controls. Both showed a moderate to strong correlation in our study, indicating that retinal thickness is reflected similarly throughout layers in an individual person. This relationship might be of relevance when interpreting GCIPL and INL in neuroinflammatory diseases, where GCIPL and INL are supposed to change in opposite directions, with GCIPL thickness reduction due to neurodegeneration (4) and INL thickening due to inflammation (12) or in response to ganglion cell loss (10).

The presented semi-automatic OCT image segmentation pipeline, SAMIRIX, provides an accessible and flexible toolbox, which can handle the entire process needed to analyze intra-retinal layer thicknesses on raw SD-OCT images. SAMIRIX is not introducing a new segmentation approach, but rather implements an existing algorithm, and extends it with processing pipelines and comfortable manual correction tools. For research use, SAMIRIX was faster compared to HEYEX, and the initial segmentation more accurate. In a few cases with severely affected eyes, initial automatic segmentation produced large errors. These cases then needed more processing time than with HEYEX, suggesting a potential in improving the initial segmentation approach. Importantly, SAMIRIX offers a transparent open-source segmentation pipeline. Of note, while we compared SAMIRIX to HEYEX, there are other commercial and academic intraretinal segmentation tools available, e.g., Orion (by Voxeleron LLC, https://www.voxeleron.com/orion/) and Iowa Reference Algorithms (by Iowa Institute for Biomedical Imaging, https://www.iibi.uiowa.edu/oct-reference).

### 4.1. Strengths and Limitations

A general problem with reporting normative data is not only different optical properties and acquisition strategies of different devices, but also different regions of interest, which are then summarized in the respective thickness or volume measurements (47). While we report 6 mm ETDRS ring thicknesses in micrometers in this study, other regions of interest can be surveyed using the accompanying *shiny* web application. Other strengths of this study are its sample size and the similar age and sex distribution in comparison to typical cohorts of autoimmune neuroinflammatory diseases. A limitation of this study is the cross-sectional design, which impairs inferences about temporal development. The most important limitation is that we included OCT scans from only one device and one scan protocol, which limits generalizability of normative data (47). Particular caution should be taken when interpreting data acquired with various instruments, since comparative studies revealed that measurements are not directly comparable between different OCT devices (48, 49) and results can even be influenced by simple software upgrades (50). Currently, SAMIRIX is only able to work with the HEYEX Vol file format (*.vol files), which is only available through specific collaborative arrangements with Heidelberg Engineering, which is a clear limitation.

Because this study was done on a Caucasian population, readers should keep in mind that our results are not necessarily applicable to other ethnicities. Grover et al. (42) found Black subjects to have a thinner retinal thickness compared to Caucasian subjects, while Tariq et al. (51) showed that average inner macula was significantly thicker in Caucasian than East Asian and South Asian children, with South Asian children having the thinnest values. These findings were also confirmed by Girkin et al. (35), which reported that Hispanic and Indian participants showed higher thickness compared to Europeans and Africans.

## Data Availability Statement

All analyses of this study are combined in a single R markdown code embedding R shiny interactive applications, which is provided alongside the raw data as Supplementary Material as well as in a public repository, in https://github.com/neurodial/am-HC-project-analysis-public.git. A copy of the source code of SAMIRIX which was used in this study and described in this paper is also provided as Supplementary Material. An up-to-date version of SAMIRIX can be found in a public repository with the address of https://github.com/neurodial/am_SAMIRIX.git. Additionally, the markdown HTML document was deployed to a server and is ready-to-use, available under http://shiny-apps.neurodial.de/shiny/am-HC-project-analysis-public/HC_traditional_params_markdown.Rmd with the username of “guest_user” and the password of “NeuroDiaL.”

## Ethics Statement

The study was approved by the ethics committee of Charité–Universitätsmedizin Berlin and conducted according to the Declaration of Helsinki in its currently applicable version. All participants gave written informed consent.

## Author Contributions

SM collected the data, developed SAMIRIX, manually corrected layers segmentation, performed statistical analysis, contributed to data interpretation, and wrote the manuscript. KG developed the OCT-Marker. NA contributed to the data collection and manual correction of layer segmentation. HZ contributed to the data collection, visual examinations, and data interpretation. SA contributed to the data collection and participant recruitment. CB contributed to the data collection and visual examinations. JM contributed to the data collection and visual examinations. FP contributed to the study management. EK contributed to the data interpretation and wrote the manuscript. AB planned and coordinated the study, contributed to statistical analysis and data interpretation, and reviewed the manuscript. All authors approved the final draft of the manuscript submitted for review and publication.

### Conflict of Interest

EK, FP, and AB are co-founders and hold shares in technology start-up Nocturne GmbH, which has commercial interest in OCT applications in neurology. EK is now an employee of Nocturne. The remaining authors declare that the research was conducted in the absence of any commercial or financial relationships that could be construed as a potential conflict of interest.
