# Supplementary material for: Normative Data and Minimally Detectable Change for Inner Retinal Layer Thicknesses Using a Semi-automated OCT Image Segmentation Pipeline
Source: Front Neurol. 2019 Nov 25;10:1117. doi: 10.3389/fneur.2019.01117 (PMC6886563; doi:10.3389/fneur.2019.01117)
Supplement: Supplementary file 1 [file Data_Sheet_1.pdf]

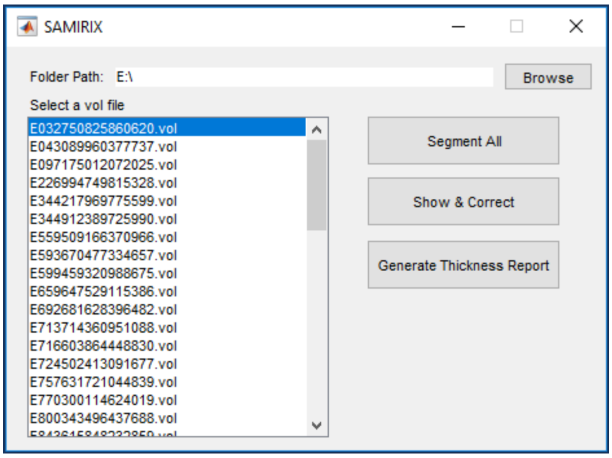

Supplementary Figure 1: A screenshot from the SAMIRIX graphical user interface.

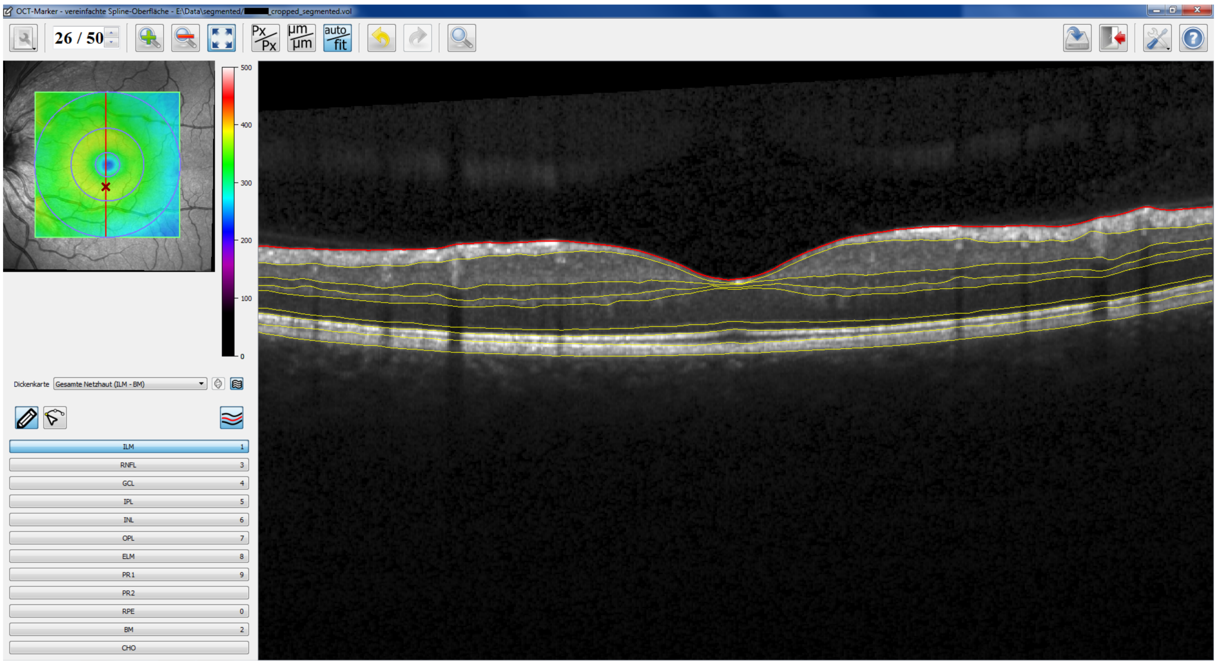

Supplementary Figure 2: A screenshot from the OCT-Marker graphical user interface.

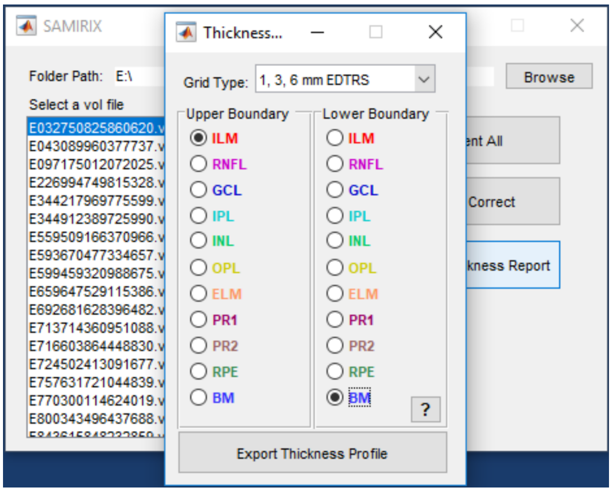

Supplementary Figure 3: A screenshot from the SAMIRIX Thickness Export graphical user interface.

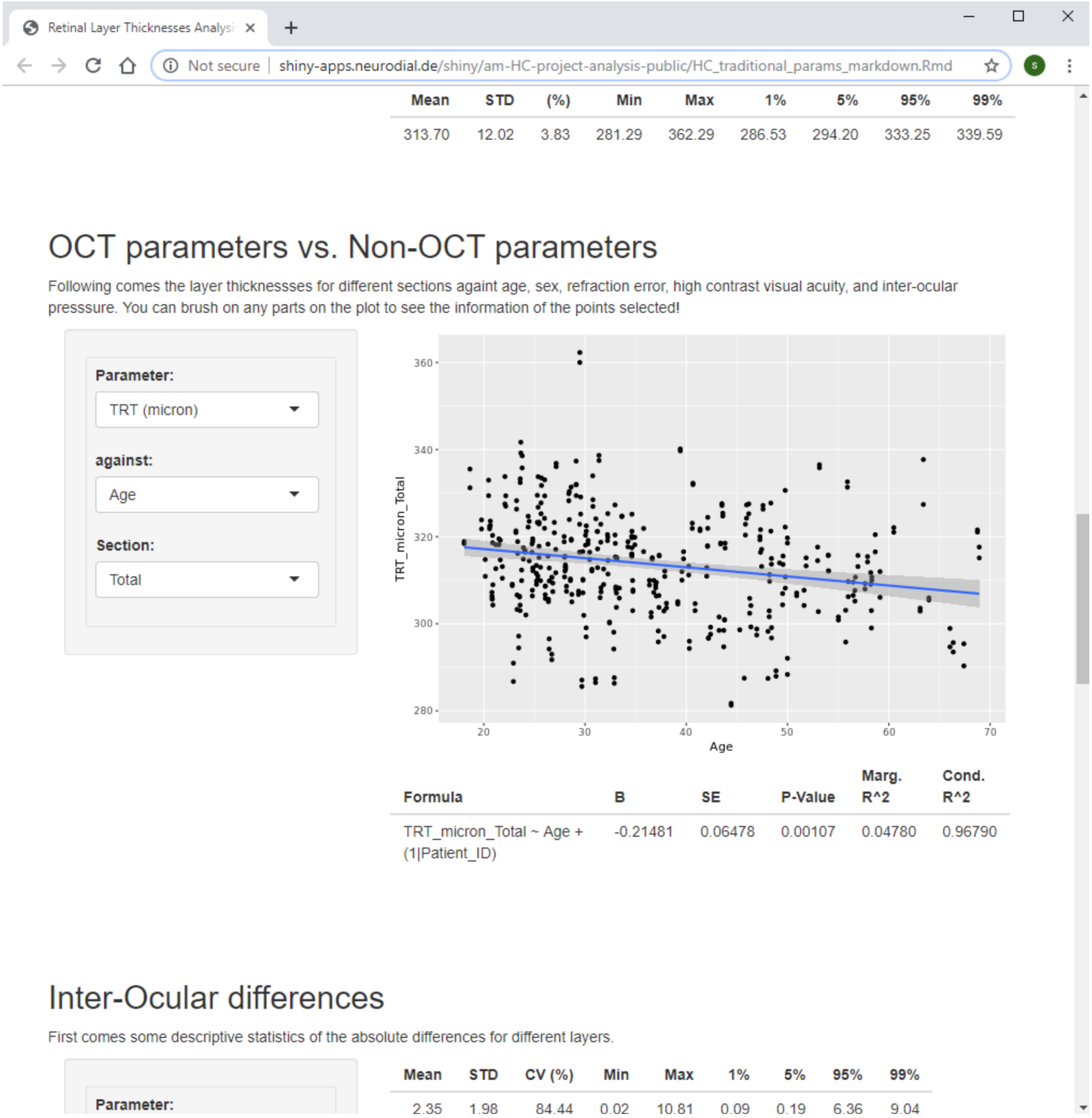

Supplementary Figure 4: A screenshot from the markdown HTML document.

Average Thickness (µm)	Mean ± SD	CV (%)	Min - Max	1st - 99th Percentile	5th - 95th Percentile
MT:					
Fovea	281.11 ± 19.04	6.77	221 - 338	239.00 - 332.46	255.00 - 316.80
Inner Nasal	354.48 ± 13.85	3.91	309 - 399	324.00 - 391.00	330.00 - 376.90
Inner Superior	353.52 ± 13.42	3.80	316 - 402	323.22 - 386.78	332.00 - 375.00
Inner Temporal	339.58 ± 13.10	3.86	309 - 382	311.22 - 374.56	319.00 - 360.00
Inner Inferior	350.81 ± 13.85	3.95	308 - 399	320.00 - 387.12	328.00 - 374.00
Outer Nasal	324.94 ± 15.10	4.65	280 - 374	291.00 - 357.78	300.10 - 350.90
Outer Superior	306.14 ± 12.76	4.17	272 - 354	279.22 - 334.56	285.00 - 327.00
Outer Temporal	289.47 ± 12.62	4.36	259 - 343	263.00 - 320.00	269.00 - 309.00
Outer Inferior	296.54 ± 12.85	4.33	262 - 348	267.22 - 322.78	274.20 - 317.00
mRNFL:					
Fovea	14.08 ± 1.27	9.02	11 - 19	11.00 - 17.00	12.00 - 16.00
Inner Nasal	27.13 ± 2.55	9.39	21 - 36	22.00 - 34.00	23.00 - 32.00
Inner Superior	31.03 ± 2.90	9.35	23 - 42	25.00 - 38.78	26.00 - 36.00
Inner Temporal	22.60 ± 1.66	7.34	17 - 27	19.00 - 26.00	20.00 - 25.00
Inner Inferior	32.50 ± 3.03	9.32	23 - 42	26.00 - 40.00	28.00 - 38.00
Outer Nasal	57.86 ± 7.20	12.44	40 - 93	44.00 - 78.56	48.00 - 71.90
Outer Superior	43.67 ± 4.61	10.57	33 - 62	34.22 - 56.78	37.00 - 52.00
Outer Temporal	24.91 ± 1.73	6.93	19 - 30	21.00 - 29.00	22.00 - 28.00
Outer Inferior	48.75 ± 6.47	13.27	32 - 76	36.22 - 66.00	40.00 - 61.00
GCIPL:					
Fovea	37.74 ± 9.18	24.32	11 - 71	19.00 - 60.78	25.00 - 56.00
Inner Nasal	95.75 ± 6.55	6.84	68 - 116	78.44 - 112.00	85.10 - 105.00
Inner Superior	95.15 ± 5.98	6.29	75 - 113	81.00 - 108.00	85.00 - 104.00
Inner Temporal	91.66 ± 6.03	6.58	71 - 107	77.00 - 106.78	82.00 - 101.00
Inner Inferior	94.88 ± 6.22	6.55	72 - 117	77.66 - 109.00	85.00 - 104.00
Outer Nasal	68.80 ± 6.19	9.00	53 - 88	56.00 - 84.78	59.00 - 79.00
Outer Superior	62.75 ± 5.08	8.09	49 - 78	51.00 - 76.78	55.00 - 71.00
Outer Temporal	68.83 ± 5.64	8.20	53 - 90	56.00 - 82.78	60.00 - 78.00
Outer Inferior	59.83 ± 5.03	8.41	48 - 74	49.00 - 73.00	52.00 - 68.00
INL:					
Fovea	21.05 ± 4.74	22.52	10 - 40	13.00 - 38.00	15.00 - 29.90
Inner Nasal	45.14 ± 3.69	8.18	34 - 57	36.00 - 54.00	39.00 - 51.90
Inner Superior	44.65 ± 3.30	7.40	35 - 54	37.00 - 52.00	39.00 - 50.00
Inner Temporal	42.16 ± 3.58	8.48	32 - 51	34.00 - 51.00	36.00 - 48.00
Inner Inferior	43.96 ± 3.08	7.01	34 - 52	37.00 - 51.00	39.00 - 49.00
Outer Nasal	35.49 ± 2.94	8.27	27 - 44	28.22 - 42.00	31.00 - 40.00
Outer Superior	33.78 ± 2.57	7.62	27 - 41	28.00 - 40.00	30.00 - 38.00
Outer Temporal	34.83 ± 2.95	8.48	27 - 44	29.00 - 41.00	30.00 - 40.00
Outer Inferior	32.30 ± 2.58	7.99	25 - 40	27.00 - 38.00	28.00 - 37.00

Supplementary Table 1: Descriptive statistics of the average thickness in different sectors of ETDRS macular map.

Abbreviations: SD: standard deviation, CV: coefficient of variation, Min: minimum, Max: maximum, MT: macular thickness, mRNFL: macular retinal nerve fiber layer, GCIPL: combined ganglion cell and plexiform layer, INL: inner nuclear layer, ETDRS: Early Treatment Diabetic Retinopathy Study.
