# Supplementary material for: Normative Data and Minimally Detectable Change for Inner Retinal Layer Thicknesses Using a Semi-automated OCT Image Segmentation Pipeline
Source: Front Neurol. 2019 Nov 25;10:1117. doi: 10.3389/fneur.2019.01117 (PMC6886563; doi:10.3389/fneur.2019.01117)
Supplement: Supplementary file 2 [file Data_Sheet_2.ZIP › Data-and-shiny/README.pdf]

README.md

Retinal Layer Thicknesses Analysis in Healthy Controls

This is a R shiny app written to analyze retina layer thicknesses (mRNFL, GCIP, etc) in healthy controls. This repository contains all the data, and the codes, which were summed up in a single RMarkdown embedding R shiny interactive applications, used for the analysis of the data for the paper **Normative data and minimally detectable change for inner retinal layer thicknesses using a semi-automated OCT image segmentation pipeline**, Motamedi et al. (Translational Neuroimaging Group (DIAL), Charité Universitätsmedizin Berlin)

Installation

The RMarkdown code was written with R version 3.5.0 and RStudio Desktop version 1.2, therefore R version 3.5.0 or higher and RStudio version 1.2 or higher is needed to successfully run this code.

The packages needed to run an RMarkdown code with Shiny apps are installed with the installation of RStudio. Additionally, these packages are needed in order to be able to run this code successfully: ***knitr, ggplot2, stringr, lmerTest, MuMIn, Hmisc, DT, pracma, ICC, dplyr, plyr, lubridate***

You can simply install all these packages by running this R code. The code installs the packages which have not been already installed.

```
list.of.packages <- c("knitr", "ggplot2", "stringr", "lmerTest", "MuMIn", "Hmisc", "DT", "pracma", "ICC", "dplyr", "pl
new.packages <- list.of.packages[!(list.of.packages %in% installed.packages()[,"Package"])]
if(length(new.packages)>0) install.packages(new.packages)
```

The version of the packages used during the development of this code is as follows: knitr v1.23, ggplot2 v3.2.0, stringr v1.4.0, lmerTest v3.1-0, MuMIn v1.43.6, Hmisc v4.2-0, DT v0.7, pracma v2.2.5, ICC v2.3.0, dplyr v0.8.1, plyr v1.8.4, lubridate v1.7.4. Although, the installation of R and RStudio with the right versions should yield to the installation of the packages with the correct versions without specifying them. R, RStudio and the packages might need administrative privileges to be installed.

After the installation of R, RStudio and the related packages, the repository needs to be cloned in the desired folder using this https URL: https://github.com/neurodial/am-HC-project-analysis-public.git, downloaded from GitHub here, or downloaded from Supplementary Material of the paper.

There is also this possibility to not use RStudio by installing the packages like Shiny which come with RStudio, but this Installation guidance is not going through this.

Usage

In order to run the RMarkdown code, please open HC_traditional_params_markdown.Rmd in RStudio, and press `Ctrl + Shift + K` on your keyboard or press `Run Document` button in RStudio. Please note that running the code might take up to several minutes.

Need Help?

In case you needed help running this code or had related questions, please contact Seyedamirhosein Motamedi (seyedamirhosein.motamedi[at]charite.de) or Dr. Alexander U. Brandt (alexander.brandt[at]charite.de). We would be delighted to help you and answer your questions!

License

Using the data and code provided in this repository without permission is prohibited. Please contact Dr. Alexander U. Brandt (alexander.brandt@charite.de) for any inquires about the data and code.
