# Supplementary material for: Normative Data and Minimally Detectable Change for Inner Retinal Layer Thicknesses Using a Semi-automated OCT Image Segmentation Pipeline
Source: Front Neurol. 2019 Nov 25;10:1117. doi: 10.3389/fneur.2019.01117 (PMC6886563; doi:10.3389/fneur.2019.01117)
Supplement: Supplementary file 3 [file Data_Sheet_3.ZIP › SAMIRIX-source-code/README.pdf]

README.md

SAMIRIX

SAMIRIX is a custom-developed intraretinal segmentation pipeline. It modularly includes import filters for OCT data, a 3rd-party segmentation algorithm, a user interface for controlling and correcting segmentation results, and batch-operations for processing multiple OCT images.

Currently, it is written in a way to work with OCTLayerSegmentation, which has been released as a package of AURA Tools on NITRC (https://www.nitrc.org/projects/aura_tools/), as the third party segmentation algorithm (OCTLayerSegmentation source code is not included).

This software was introduced and described in details in a paper **Normative data and minimally detectable change for inner retinal layer thicknesses using a semi-automated OCT image segmentation pipeline**, Motamedi et al. (Translational Neuroimaging Group (DIAL), Charité Universitätsmedizin Berlin).

Installation

In order to run SAMIRIX, MATLAB v2016b or later installed on a Windows PC is needed. Additionally, in order to use SAMIRIX with all its features, the software below should be downloaded and placed in the "Samirix" directory:

1. OCTLayerSegmentation2.11 (https://www.nitrc.org/frs/?group_id=905)
2. OCT-Marker (octmarker64 https://github.com/neurodial/OCT-Marker/releases)

After placing "octmarker64" and "OCTLayerSegmentation2.11" folders in the "Samirix" folder, the main folder (the folder which contains the LICENSE.txt and README file) and all its subfolders have to added to MATLAB Path (by right clicking on the main folder in the Current Folder layout in MATLAB and selecting Add to Path > Selected Folders and Subfolders).

Usage

You can run SAMIRIX by typing "Samirix_GUI" and pressing Enter in Command Window or by openning "Samirix_GUI.m" and pressing the Run Button (or F5).

In order to understand how SAMIRIX functions, please read the sections describing SAMIRIX in the paper.

Please note that it is a common mistake to open Samirix_GUI.fig instead of opening and running Samirix_GUI.m, which should be avoided.

Contact

Please contact Dr. Alexander U. Brandt (alexander.brandt@charite.de) for any inquires about this software.

License

Please read LICENSE.txt.
