# Supplementary figures and images for: Normative Data and Minimally Detectable Change for Inner Retinal Layer Thicknesses Using a Semi-automated OCT Image Segmentation Pipeline
Source: Front Neurol. 2019 Nov 25;10:1117. doi: 10.3389/fneur.2019.01117 (PMC6886563; doi:10.3389/fneur.2019.01117)

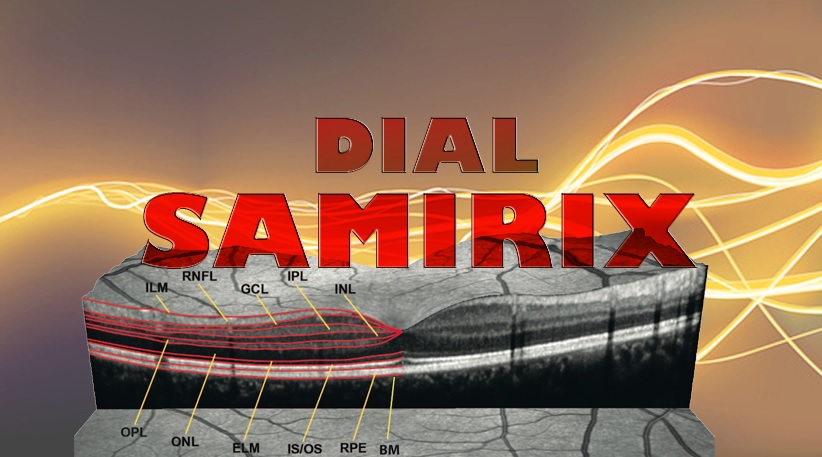

Supplement: Supplementary file 3 [file Data_Sheet_3.ZIP › SAMIRIX-source-code/Samirix/samirix.jpg]

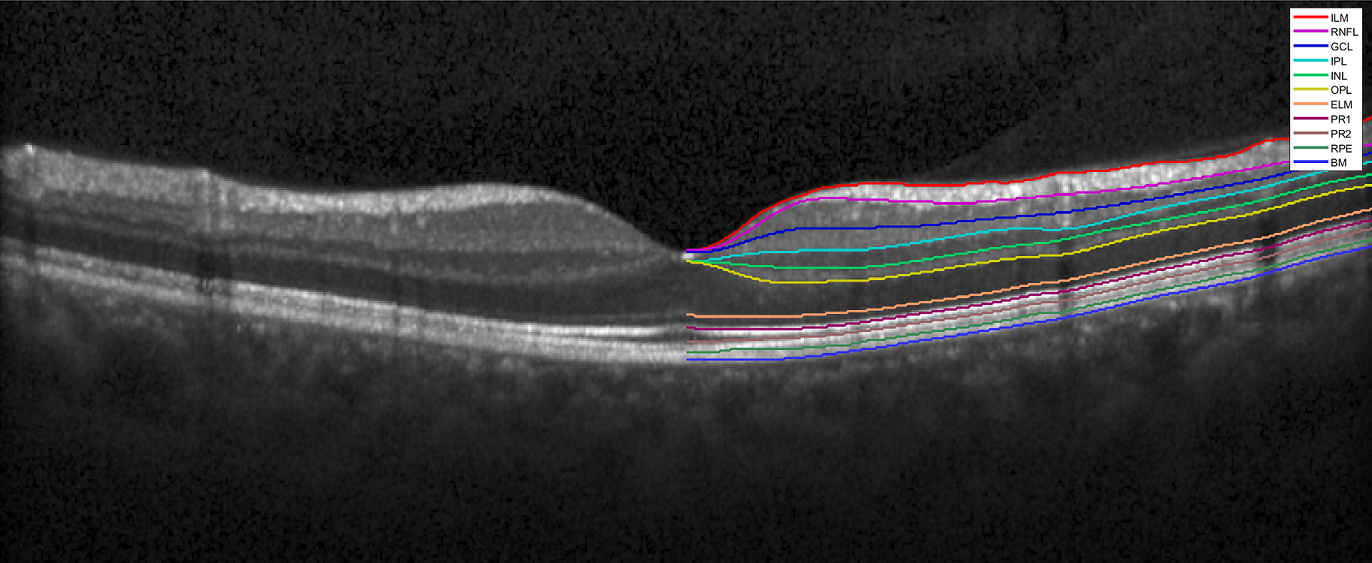

Supplement: Supplementary file 3 [file Data_Sheet_3.ZIP › SAMIRIX-source-code/Samirix/thicknessMapHelp - Kopie.png]
